# Widespread Occurrence of Two Carbon Fixation Pathways in Tubeworm Endosymbionts: Lessons from Hydrothermal Vent Associated Tubeworms from the Mediterranean Sea

**DOI:** 10.3389/fmicb.2012.00423

**Published:** 2012-12-14

**Authors:** Vera Thiel, Michael Hügler, Martina Blümel, Heike I. Baumann, Andrea Gärtner, Rolf Schmaljohann, Harald Strauss, Dieter Garbe-Schönberg, Sven Petersen, Dominique A. Cowart, Charles R. Fisher, Johannes F. Imhoff

**Affiliations:** ^1^GEOMAR, Helmholtz Centre for Ocean Research KielKiel, Germany; ^2^Water Technology Center KarlsruheKarlsruhe, Germany; ^3^Institut für Geologie und Paläontologie, Westfälische Wilhelms-Universität MünsterMünster, Germany; ^4^Institut für Geowissenschaften, Christian – Albrechts – Universität KielKiel, Germany; ^5^Mueller Laboratory, Department of Biology, The Pennsylvania State UniversityUniversity Park, PA, USA

**Keywords:** hydrothermal vent, vestimentiferan tubeworm, carbon fixation, endosymbiont, *acl* gene, cbbM gene, Lamellibrachia, Mediterranean Sea

## Abstract

Vestimentiferan tubeworms (siboglinid polychetes) of the genus *Lamellibrachia* are common members of cold seep faunal communities and have also been found at sedimented hydrothermal vent sites in the Pacific. As they lack a digestive system, they are nourished by chemoautotrophic bacterial endosymbionts growing in a specialized tissue called the trophosome. Here we present the results of investigations of tubeworms and endosymbionts from a shallow hydrothermal vent field in the Western Mediterranean Sea. The tubeworms, which are the first reported vent-associated tubeworms outside the Pacific, are identified as *Lamellibrachia anaximandri* using mitochondrial ribosomal and cytochrome oxidase I (COI) gene sequences. They harbor a single gammaproteobacterial endosymbiont. Carbon isotopic data, as well as the analysis of genes involved in carbon and sulfur metabolism indicate a sulfide-oxidizing chemoautotrophic endosymbiont. The detection of a hydrogenase gene fragment suggests the potential for hydrogen oxidation as alternative energy source. Surprisingly, the endosymbiont harbors genes for two different carbon fixation pathways, the Calvin-Benson-Bassham (CBB) cycle as well as the reductive tricarboxylic acid (rTCA) cycle, as has been reported for the endosymbiont of the vent tubeworm *Riftia pachyptila*. In addition to RubisCO genes we detected ATP citrate lyase (ACL – the key enzyme of the rTCA cycle) type II gene sequences using newly designed primer sets. Comparative investigations with additional tubeworm species (*Lamellibrachia luymesi*, *Lamellibrachia* sp. 1, *Lamellibrachia* sp. 2, *Escarpia laminata, Seepiophila jonesi*) from multiple cold seep sites in the Gulf of Mexico revealed the presence of *acl* genes in these species as well. Thus, our study suggests that the presence of two different carbon fixation pathways, the CBB cycle and the rTCA cycle, is not restricted to the *Riftia* endosymbiont, but rather might be common in vestimentiferan tubeworm endosymbionts, regardless of the habitat.

## Introduction

Vestimentiferan tubeworms are often dominant members of chemosynthetic communities present at reduced environments such as hydrothermal vents and cold seeps (Vrijenhoek, 2010). So far, hydrothermal vent-associated tubeworms have not been found outside the Pacific. In contrast, seep-associated tubeworms have been found in the Gulf of Mexico (GoM), the Mediterranean Sea, and the margins of the Atlantic Ocean (Cordes et al., 2009; Vrijenhoek, 2010).

**Figure 1:**
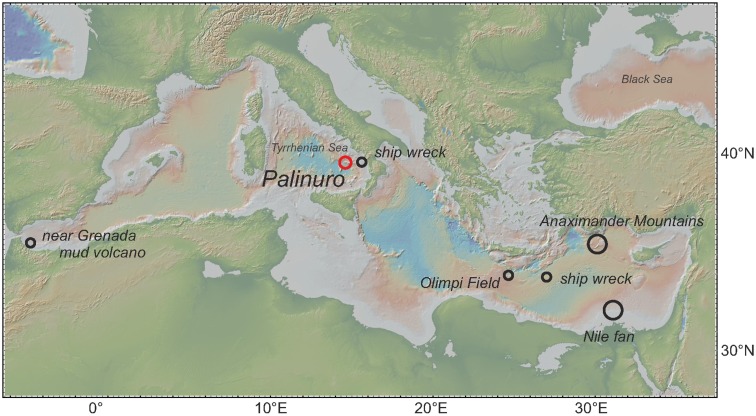
**Map with locations of vestimentiferan tubeworms of the genus *Lamellibrachia* described from previous studies (black circles) and this study (red circle) within the Mediterranean Sea**.

The Mediterranean Sea is the world’s largest enclosed sea, and represents a hot spot of biodiversity with a considerable number of endemic species (Myers et al., 2000). Its only connection to the Atlantic Ocean is the narrow and shallow Strait of Gibraltar, which is the sole route for exchange of propagules between these two water bodies. The only vestimentiferan tubeworms documented to date in the Mediterranean Sea belong to the genus *Lamellibrachia* and specimens from several Mediterranean mud volcanoes were recently described as the new species *Lamellibrachia anaximandri* (Southward et al., 2011). The genus *Lamellibrachia* has a worldwide distribution, and occurs in several types of chemosynthetic environments from the shallow to the deep-sea (e.g., Kojima et al., 2002). Within the Mediterranean Sea, *Lamellibrachia* spp. have been discovered in the vicinity of mud volcanoes in the Alboran Sea at 572 m depth (Hilário et al., 2011), from several mud volcanoes in the Eastern Mediterranean Sea at a depth of about 3,000 m (Olu-Le Roy et al., 2004; Bayon et al., 2009; Duperron et al., 2009; Southward et al., 2011) and also from two sunken ship wrecks in the Eastern and Western Mediterranean (Hughes and Crawford, 2008; Gambi et al., 2011; Figure 1).

Hydrocarbon seep communities in the GoM were among the first seep communities to be discovered, are extensively studied, and have a high diversity of tubeworm species (Kennicutt et al., 1985; Miglietta et al., 2010). The Louisiana Slope in the northern GoM area extends from the continental shelf to the salt deformation edge of the Sigsbee Escarpment, and ranges from about 300 to 3,000 m in depth. This area is home to at least six known morphospecies of vestimentiferan tubeworms (Miglietta et al., 2010), including the most commonly studied seep tubeworms, *Lamellibrachi luymesi* (van der Land and Nørrevang, 1975), and *Seepiophila jonesi* (Gardiner et al., 2001).

In contrast to the well-known hydrothermal vent tubeworm *Riftia pachyptila*, which inhabits hard substrate in hot sulfidic environments, members of the genus *Lamellibrachia* live in sedimented areas and are most common in cold seep environments. Seep habitats are generally much less dynamic than vent habitats and may be stable for centuries (Fisher et al., 1997). Compared to vent environments, emanating seep fluids are cooler, often enriched in methane and concentrations of dissolved sulfide may be quite low (Southward et al., 2011). *Lamellibrachia* tubeworms can obtain sulfide from the underlying sediments using the buried, permeable posterior region of the tube termed the “root” (Julian et al., 1999; Freytag et al., 2001). Since *Lamellibrachia*, like other siboglinid polychetes, lack a digestive tract, they are dependent on their endosymbionts for nutrition. Sulfide is transported via hemoglobin molecules in the blood to the trophosome, a large organ that harbors dense populations of gammaproteobacterial endosymbionts (reviewed by Childress and Fisher, 1992). These endosymbionts oxidize the sulfide to obtain energy and reducing power for autotrophic carbon fixation. A portion of the synthesized organic matter serves in turn as energy source for the host tubeworm (Bright et al., 2000; Stewart and Cavanaugh, 2006). *Lamellibrachia* spp. are not only found at cold seeps, but also at sediment covered hydrothermal sites, e.g., *Lamellibrachia barhami* along the Juan de Fuca Ridge (Juniper et al., 1992), *Lamellibrachia columna* near hydrothermal vents in the Lau Basin in the southwest Pacific (Southward, 1991) and *Lamellibrachia satsuma* at hydrothermal sites off southern Japan (Miake et al., 2006). Even though the so-called “vent” and “seep” tubeworm genera are clearly specialized for their preferred *in situ* conditions, they have been found at the same site, sometimes occurring only meters apart, e.g., the seep tubeworm *L. barhami* and the vent species *Ridgeia piscesae* at Middle Valley in 2,400 m depth in the northeast Pacific Ocean (McMullin et al., 2003).

Vestimentiferan endosymbionts form a monophyletic cluster within the gammaproteobacteria. They have been shown to cover very large geographic ranges, with nearly identical 16S rRNA in hosts separated by thousands of kilometers. Within the endosymbiont cluster four different groups (one “vent”-group, three “seep”-groups) are distinguishable. So-called “vent” endosymbionts appear to be specific for vent vestimentiferan hosts (e.g., *Riftia*, *Tevnia*, *Ridgeia*, and *Oasisia*), while three different 16S rRNA gene clusters (groups 1–3), possibly representing different strains, were found only in “seep” vestimentiferans (Nelson and Fisher, 2000; McMullin et al., 2003). Site depth has been postulated to be a factor in defining which of the three endosymbiont strains is found in a particular “seep” host, with “group 3” occurring only in shallow water host specimens (McMullin et al., 2003).

The best-studied tubeworm endosymbiont is *Candidatus* Endoriftia persephone, the gammaproteobacterial endosymbiont of the vent-associated tubeworm *Riftia pachyptila*. Its metabolic capacities have been subject of detailed metagenomic, proteomic, and enzymatic studies (Felbeck, 1981; Felbeck et al., 1981; Markert et al., 2007, 2011; Robidart et al., 2008; Gardebrecht et al., 2012). *Candidatus* Endoriftia persephone is a sulfide-oxidizing chemoautotroph. Sulfide is oxidized to sulfate via sulfite and adenosine phosphosulfate (APS). The enzymes involved in the so-called APS pathway are dissimilatory sulfite reductase (DsrAB, working in reverse as sulfide oxidase), APS reductase (AprAB), and ATP sulfurylase (Markert et al., 2007, 2011). Quite surprising is the presence of two alternative carbon fixation pathways in the *Riftia* endosymbiont, the Calvin–Benson–Bassham (CBB) cycle as well as the reductive tricarboxylic acid (rTCA) cycle (Felbeck, 1981; Markert et al., 2007). Both pathways show unique features. The CBB cycle seems more energy-efficient due to modified enzyme equipment (Markert et al., 2011; Gardebrecht et al., 2012; Kleiner et al., 2012) while the rTCA cycle harbors a novel type of ATP citrate lyase (Hügler and Sievert, 2011). In contrast to the *Riftia* endosymbiont there are no genomic, proteomic or metabolomic studies of the endosymbiont(s) of *Lamellibrachia* spp.

This study reports the recovery of vestimentiferan tubeworms from the Palinuro volcanic complex, a submarine volcano in the Tyrrhenian Sea (Western Mediterranean Sea), north of Sicily (Figure 1). The Palinuro complex is part of the active Aeolian Island Arc and consists of several volcanic edifices aligned over a strike length of 55 km (Petersen et al., 2008; Passaro et al., 2010). The volcanic complex is up to 25 km wide at its base and its shallowest portion rises from 3,000 m to a water depth of less than 100 m. Iron and manganese-bearing precipitates were first documented at Palinuro by Kidd and Ármannson (1979) providing the first evidence for hydrothermal activity in the area. Hydrothermal sulfides were described by Minniti and Bonavia (1984) and Puchelt and Laschek (1987) within sediment sampled from the most westerly summit of Palinuro. The discovery of living vestimentiferan tubeworm colonies on top of the main volcanic edifices in this western summit in 2006 as well as temperatures of up to 60°C in sediment cores recovered from the seafloor indicated that active hydrothermal venting was taking place at the time although black smoker style venting has not been observed (Petersen et al., 2008; Monecke et al., 2009). Two colonies of these tubeworms were sampled in spring 2011.

**Figure 2:**
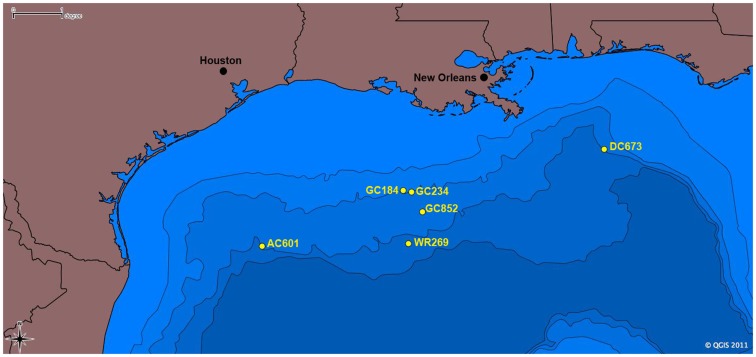
**Map of sampling locations for tubeworm specimens in the Gulf of Mexico included in this study**. Scale is in degrees longitude; 1° = 111.12 km.

We describe the results from detailed analyses of the Palinuro tubeworms and their endosymbionts, which are the first reported vent-associated tubeworms outside the Pacific Ocean. For comparison, several seep tubeworm species from the GoM were also analyzed (Figure 2), providing deeper insights into the geographic dispersal, phylogeny, and metabolic potential of tubeworms and their endosymbionts.

## Materials and Methods

### Sampling site, sample collection, and processing of Palinuro tubeworms

Vestimentiferan tubeworm specimens were retrieved from two different colonies termed colony #1 and #2 on the western summit of the Palinuro volcanic complex (Mediterranean Sea, 39°32.44′N, 14°42.38′E, depth: 630 m) during the Pos412 cruise of R/V *Poseidon* in spring 2011. Sampling was conducted using a Mohawk-type remotely operated vehicle (ROV) supplied by Oceaneering Inc. (Aberdeen, UK) fitted with a robotic arm. Locations of the tubeworm collections are given in Table 1.

**Table 1:** **Position of tubeworm colonies sampled during R/V POSEIDON cruise Pos412 at Palinuro volcanic complex, Tyrrhenian Sea and temperatures measured within colonies**.

Tubeworm colony no.	Station no.	Geographical position (TMS; ROV)	Depth (m)	Temperature (°C)
Colony #1	231-1	39°32.45′N/014°42.41′E	634	Top: 14.2
				Center: 19.4
				Base: 15.9
Colony #2	241-3	39°32.427′N/014°42.384′E	630	Top: 15.6
				Center: 14.7
				Base: 14.2

The ROV was also equipped with a fluid sampling system (Kiel *in situ* pumping system, KIPS, Garbe-Schönberg et al., 2006) capable of acquiring four 550 mL water samples per dive with *in situ* filtration. Parallel to the sampling nozzle was a temperature probe attached to a data logger. Fluids and temperatures around the colonies were sampled using KIPS and the temperature probe by maneuvering the ROV’s robotic arm into the fluid in close proximity to the living tubeworms. Live tubeworms were sampled using the ROV’s robotic arm. Immediately after tubeworm sampling, the dive was terminated, and the ROV was recovered. Upon recovery, the tubeworms obtained from colony #1 were put into sterile Petri dishes using sterile tweezers followed by dissection with a sterile scalpel. The animal was then separated from the tube; subsamples were recovered from vestimentum (host tissue free of symbionts) and trophosome (endosymbiont) tissue, and then were stored at −20°C until further molecular analysis. Samples from colony #2 were immediately stored at −20°C until further processing in the home laboratory. As the tubeworms from colony #2 exhibited several morphotypes, these were stored in separate vials.

### Gulf of Mexico tubeworm sample collection and preparation

Gulf of Mexico vestimentiferan tubeworms were sampled from hydrocarbon seep sites during several research cruises between 1997 and 2011 (Table 2; Figure 2). *Lamellibrachia luymesi* and *Seepiophila jonesi* were collected using the Johnson Sea Link submersible from two sites on the Upper Louisiana Slope from about 540 m depth. *Lamellibrachia* sp. 1 and *Lamellibrachia* sp. 2 as well as *Escarpia laminata* were collected from three sites on the Lower Louisiana Slope ranging in depth from 1,975 to 2,604 m. While on board the research vessel, tubeworms were dissected and vestimentum (host tissue free of symbionts) and trophosome (endosymbiont) tissue was preserved at −80°C or in 95% ethanol solution. All tissue samples were transported to the Pennsylvania State University where whole genomic DNA was obtained using a modified version of the high salt extraction protocol and ethanol precipitation as in Liao et al. (2007). Isolated DNA is currently stored at −80°C at the Pennsylvania State University.

**Table 2:** **Sample identity, geographic origin, and gene sequences accession numbers of tubeworm specimens and their endosymbionts included in this study**.

Sample name	Species	Region	Site	Depth (m)	Date collected	Latitude	Longitude	Endosymbiont 16S rRNA	Endosymbiont cbbM	Endosymbiont ACL	Host mt16S rRNA	Reference
Pos412-B1_L1-7	*Lamellibrachia anaximandri*	Mediterranean Sea, Palinuro Seamount	Pos412-231	634	4/27/2011	39°32.450′N	015°42.410′E	HE983342-8	HE978225	HE978244	HE974472	This study
Pos412-B2_L1-4	*Lamellibrachia anaximandri*	Mediterranean Sea, Palinuro Seamount	Pos412-242	630	4/29/2011	39°32.427′N	015°42.384′E	HE983349-52	HE978226-8	HE978245-6	HE974473	This study
DC673_1211	*Lamellibrachia* sp. 1	Gulf of Mexico, DeSoto Canyon	DC673	2604	10/30/2006	28°18.603′N	087°18.643′W	HE983327	HE978212	HE978229	HE974464	This study
DC673_1209	*Lamellibrachia* sp. 2	Gulf of Mexico, DeSoto Canyon	DC673	2604	10/30/2006	28°18.603′N	087°18.643′W	HE983328	HE978213	HE978230	HE974465	This study
DC673_1170	*Escarpia laminata*	Gulf of Mexico, DeSoto Canyon	DC673	2604	10/29/2006	28°18.603′N	087°18.643′W	HE983329	HE978214	HE978231	HE974466	This study
AC601_E6	*Escarpia laminata*	Gulf of Mexico, Alaminos Canyon	AC601	2339	5/27/2002	26°23.365′N	094°30.880′W	HE983330	HE978215	HE978232	GU068203	Miglietta et al. (2010), This study
AC601_L1	*Escarpia laminata*	Gulf of Mexico, Alaminos Canyon	AC601	2339	5/27/2002	26°23.365′N	094°30.880′W	HE983331-2	HE978216	HE978233-4	HE974467	This study
WR269_E10	*Escarpia laminata*	Gulf of Mexico, Walker Ridge	WR269	1975	5/25/2002	26°40.672′N	091°39.691′W	HE983333	HE978217	HE978235	HE974468	This study
GC852_L4	*Lamellibrachia* sp. 1	Gulf of Mexico, Green Canyon	GC852	1437	5/22/2002	27°05.768′N	091°09.897′W	HE983334	HE978218	HE978236	GU068242	Miglietta et al. (2010), This study
GC852_L1	*Lamellibrachia* sp. 2	Gulf of Mexico, Green Canyon	GC852	1437	5/22/2002	27°05.768′N	091°09.897′W	HE983335	HE978219	HE978237	HE974469	This study
GC852_L5	*Lamellibrachia* sp. 1	Gulf of Mexico, Green Canyon	GC852	1437	5/22/2002	27°05.768′N	091°09.897′W	HE983336	HE978220	HE978238	HE983353	This study
AC601_L20	*Lamellibrachia* sp. 2	Gulf of Mexico, Alaminos Canyon	AC601	2335	5/30/2002	26°23.548′N	094°30.849′W	HE983337-8	HE978221	HE978239-40	HE974470	This study
GC234_4587	*Seepiophila jonesi*	Gulf of Mexico, Green Canyon	GC234	527	2003	27°26.839′N	091°08.061′W	HE983339	HE978222	HE978241	HE974471	This study
GC184_L9	*Lamellibrachia luymesi*	Gulf of Mexico, Green Canyon	GC184	540	1995	27°28.171′N	091°18.265′W	HE983340	HE978223	HE978242	GU068216	Miglietta et al. (2010), This study
GC234_L7	*Lamellibrachia luymesi*	Gulf of Mexico, Green Canyon	GC234	546	1995	27°26.847′N	091°07.986′W	HE983341	HE978224	HE978243	GU068238	Miglietta et al. (2010), This study

### DNA extraction, PCR amplification, cloning, and sequencing

Genomic DNA was extracted from the trophosome and vestimentum tissues of the vestimentiferan tubeworms. The tubeworms were dissected and washed three times in 0.2 μm filtered seawater prior to DNA extraction. DNA of Mediterranean tubeworm samples was isolated using the MoBio Power Biofilm Kit (Mo Bio Laboratories, Carlsbad, CA, USA) according to the protocol provided. DNA of GoM tubeworm samples was extracted following the protocol of Liao et al. (2007). Cytochrome c oxidase I (COI) genes, mitochondrial and bacterial ribosomal (16S rRNA) genes, as well as *cbbM* and ACL type II genes were analyzed from all individuals. Further functional genes and eukaryotic ribosomal (18S rRNA) genes were analyzed from one individual of colony #1 from the Palinuro volcanic complex.

For all gene amplifications of Mediterranean samples PCR reactions were conducted using Ready-To-Go PCR Beads (GE Healthcare, Munich, Germany) in a total volume of 25 μL. PCR from GoM samples were conducted using 1 U BioBasic TaqPolymerase (BioBasic Inc., Markham, ON, Canada) and 1× Thermopol Buffer (NEB Inc., USA) in a total volume of 50 μL. If not stated otherwise 10 pmol of each primer and 100 ng template DNA was used. For all amplifications, initial denaturation was 2 min at 94°C, final annealing was 1 min at annealing temperature, and final elongation 5 min at 72°C. For the cycles denaturation was 40 s at 94°C, annealing duration 40 s at the respective annealing temperature and elongation was 1 min at 72°C. If not stated otherwise, 35 PCR cycles were applied. Fragments of the tubeworms’ 18S rRNA and mitochondrial 16S rRNA as well as COI genes were amplified using the (i) primer pairs 5′-start (5′-GGT TGA TCC TGC CAG-3′) and 1753rev (5′-GCA GGT TCA CCT ACG G-3′) targeting the 18S rRNA gene (30 cycles, 50°C annealing temperature), (ii) primer pair 16Sar/16Sbr (Palumbi et al., 2002) targeting the mitochondrial 16S rRNA gene (30 cycles, 50°C annealing temperature), and (iii) primers LCO 1490 (5′-GGT CAA CAA ATC ATA AAG ATA TTG G-3′) and HCO 2198 [5′-TAA ACT TCA GGG TGA CCA AAA AAT CA-3′; 40 cycles, 47°C annealing temperature (Folmer et al., 1994)] using DNA extracted from the symbiont-free vestimentum tissue. Gene fragments of the endosymbiont were amplified using DNA extracted from trophosome tissue as the template. Bacterial 16S rRNA gene fragments were amplified in a 30 cycle PCR at an annealing temperature of 50°C with the general bacterial primer set 27F and 1390R (Palinuro samples; 5′-GAC GGG CRG TGT GTA CAA-3′) or 1492R (GoM samples; Lane, 1991). Amplification for fragments of *dsrA* and *aprA* genes was performed using the primer sets r*dsrA*240F/r*dsrA*403R and *aps*1F/*aps*4R, respectively (Meyer and Kuever, 2007b; Lavik et al., 2009). Fragments of *soxB* were amplified using the primers *soxB*432F/*soxB*1446B [10 cycles with 55°C annealing temperature and 25 cycles with 47°C annealing temperature (Petri et al., 2001)]. For the amplification of fragments of the genes coding for the large subunit of RubisCO form I and II, the primer sets *cbbL*F/*cbbL*R and *cbbM*F/*cbbM*R were used [both include two initial cycles of 2 min annealing at 37°C and 3 min elongation at 72°C, as well as additional 35 cycles of 53 and 58°C annealing temperature for *cbbL* and *cbbM* respectively (Campbell and Cary, 2004)]. A fragment of the large subunit of the putative type II ATP citrate lyase gene was amplified using the newly designed primer *acl2*F1 (5′-CGT CGC CAA GGA AGA GTG GTT C-3′) and *acl2*R1 (5′-GGC GAT GGC CTC AAA GCC GTT-3′) in a 30 cycle PCR with annealing temperatures of 45–56°C (gradient). Fragments of the hydrogen uptake hydrogenase gene *hupL* were amplified with the primer set HUPLX1/HUPLW2 (Csaki et al., 2001). A fragment of the *norCB* gene for nitric oxide reductase subunits C and B was amplified using the primer set *nor*C21mF and *nor*B6R (Tank, 2005) in a 35 cycle PCR using annealing temperatures of 60–50°C (10 touchdown cycles 60°C/−1°C, 25 cycles of 50°C).

Additional primer pairs used in this study include: F2/R5 and 892F/1204R for the two subunits of ATP citrate lyase (Campbell et al., 2003; Hügler et al., 2005); MxaF1003, MxaR1555, MxaR1561 for methanol dehydrogenase gene *mxaF* (Neufeld et al., 2007; Kalyuzhnaya et al., 2008); *mmoXA/mmoXB* for genes encoding the conserved alpha-subunit of the hydroxylase component of the cytoplasmatic soluble methane monooxygenase (sMMO; Auman et al., 2000); and A189F/MB661R for the particulate methane monooxygenase (pMMO) genes present in methanotrophs (Costello and Lidstrom, 1999).

All PCR products were purified via gel extraction using QIAquick gel extraction kit (QIAgen, Hilden, Germany) for Mediterranean samples, and BioBasic EZ-10 spin columns (BioBasic Inc., Markham, ON, Canada) for GoM samples respectively, and either directly sequenced by Sanger sequencing (18S rRNA gene fragments, COI, and functional gene fragments) or cloned into pCR4-TOPO vectors with the TOPO-TA cloning kit (Invitrogen, Carlsbad, CA, USA) as described by the manufacturer before sequencing (16S rRNA gene fragments). Sequencing was conducted using amplification primers and additional internal primers in the case of 16S rRNA genes (342F, 534R; Muyzer et al., 1996); 790F (Thiel et al., 2007). Amplification and sequencing of clones was conducted using vector specific primers M13 forward and M13 reverse (PCR) and T3 and T7 (sequencing), respectively. Sanger sequencing was performed using the BigDye Terminator v1.1 sequencing kit in a 3730xl DNA Analyzer (Applied Biosystems, Carlsbad, CA, USA) as specified by the manufacturer. Sequencing was conducted by the Institut für Klinische Molekularbiologie (IKMB), Universitäts-Klinikum Schleswig-Holstein (UK-SH), Kiel, Germany and the sequencing core facility at The Pennsylvania State University, University Park, PA, USA.

### Phylogenetic analysis

All sequences were edited with ChromasPro c.c1.33 and compared to the NCBI database using BLAST (Altschul et al., 1997). Functional gene nucleotide sequences were also compared with the non-redundant protein sequence database using the blastx algorithm. The endosymbiont 16S rRNA gene sequences were aligned with the ARB software (www.arb-home.de) using the ARB FastAligner utility (Ludwig et al., 2004). The sequence alignment was manually refined based on known secondary structures. Sequences of functional genes as well as mitochondrial rRNA genes were aligned using Clustal X (Thompson et al., 1997) and manually adjusted using BioEdit (Hall, 1999). Maximum Likelihood based trees and 100 bootstrap replicates were constructed using PhyML (Guindon and Gascuel, 2003). In order to verify the tree topology, further phylogenetic analyses using Neighbor-Joining and Maximum-Parsimony algorithms were conducted using MEGA5 (Tamura et al., 2011).

### Microscopy

The morphology of endosymbiotic bacteria in trophosome tissue of the tubeworms was examined using light microscopy and scanning electron microscopy (SEM). Samples for light microscopy were prepared by removing small pieces of tissue from different parts of the trophosome and subsequent squeezing preparation in a drop of particle-free seawater and examined under 100-fold magnification using a Zeiss Axiophot Epifluorescence Microscope.

Samples for SEM were prepared by disrupting small trophosome samples in 0.2 μm filtered seawater, and then concentrated by filtration on to 0.2 μm polycarbonate membrane filters followed by dehydration through ascending concentrations of ethanol. Subsequently, the samples were critical-point-dried using a Balzers CPD 030 and CO_2_ as a transition medium. The filters were sputter-coated with gold-palladium using a Balzers SCD 004 and observed with a Zeiss DSM 940 electron microscope.

### Fluid chemistry

After recovery of the ROV Mohawk all KIPS fluid samples were immediately transferred to the onboard ship lab and sub-sampled for subsequent analyses. Both pH and Eh of the fluids were determined immediately after sub-sampling using electrochemical techniques after calibration with certified standards. Dissolved oxygen was determined using standard Winkler titration protocols modified for small volumes. The concentration of dissolved sulfide was determined in 1 mL aliquots using a zinc acetate gelatin solution, which precipitates the dissolved sulfide as colloidal zinc sulfide. Subsequently, the color agent, *N,N*-dimethyl-1,4-phenylenediamine-dihydrochloride, and a catalyst, iron chloride solution, were added to form methylene blue (Cline, 1969). After 1 h, the solutions were measured photometrically at a wavelength of 660 nm using a Genesys Spectra 10 spectrophotometer. The detection limit was 1 μmol/L. Potential oxidation of dissolved hydrogen sulfide during sampling and sample recovery cannot be ruled out, but is likely minimal. Nonetheless, hydrogen sulfide concentration data given in this paper should be considered minimum values. Aliquots for the analysis of nutrients were stored in polypropylene bottles, sealed, and stored in the dark at 4°C until analysis. Aliquots for cation and trace element analysis were pressure-filtrated through 0.2 μm Nucleopore polycarbonate (PC) membrane filters using Sartorius PC filtration units and high purity nitrogen. Samples were acidified with subboiled nitric acid to pH <2 and stored in perfluoralkoxy (PFA) bottles until analysis. Multielement analysis for major ion composition (Cl, B, Si, Na, K, Ca, Mg, Fe, Mn) of the water samples was performed with a SPECTRO Ciros SOP ICP-OES spectrometer after 10-fold dilution and using Y for internal standardization. Trace elements (As, Li, W) were determined by ICP-MS (Agilent 7500 cs at University of Kiel) after 12.5-fold dilution using both In and Re for internal standardization. Certified reference materials NIST1643e, NASS-5, and IAPSO were used for validation and accuracy checks.

### Carbon isotope signature

The organic carbon isotopic composition of tubeworm tissue (δ^13^C_ORG_) was determined via continuous flow EA-IRMS using an elemental analyzer interfaced to a ThermoFinnigan Delta Plus isotope ratio mass spectrometer. Briefly, about 40–60 μg of freeze-dried worm tissue was weighed in tin capsules, combusted to CO_2_, and chromatographically purified carbon dioxide was transferred to the mass spectrometer in a He gas stream. Results are reported in the standard delta notation as per mil difference to the Vienna Pee Dee Belemnite. Sample measurements were done in duplicate, and analytical performance was monitored with international reference materials (USGS 24; USGS 40) and lab standards (anthracite; brown coal) and the reproducibility was generally better than ±0.15‰.

The carbon isotopic composition of dissolved inorganic carbon (δ^13^C_DIC_) from vent fluids was measured using a Thermo Finnigan Gas Bench coupled to a Thermo Finnigan Delta Plus XL. Briefly, 0.5–1.0 mL of hydrothermal fluid was injected into an exetainer that contained phosphoric acid, liberating DIC as carbon dioxide. Prior to sample injection, the exetainer was flushed with helium. CO_2_ was flushed from the exetainer with a stream of helium and injected into the mass spectrometer. Results are reported in the standard delta notation as per mil difference to the Vienna Pee Dee Belemnite. Sample measurements were done in duplicate, and analytical performance was monitored with a sodium carbonate lab standard.

### Nucleotide sequence accession numbers

The sequence data have been submitted to EMBL/GenBank/DDBJ databases under accession numbers HE9744464-85, HE978212-46, and HE983327-53.

## Results

### Biogeochemical characterization of the tubeworm habitat at Palinuro

For the present study, two colonies of vestimentiferan tubeworms as well as their biogeochemical environment were sampled by means of a ROV. The tubeworms occurred within a sediment-filled depression at the western summit of the Palinuro volcanic complex in water depths of around 630 m and formed small bushes, up to 1 m^2^ in diameter, mainly on sedimented surfaces but some patches also occurred in areas where volcanic rocks were present at the seafloor. Frequently, shimmering water was observed rising above the tubeworm colonies suggesting active fluid emanation. The first colony (colony #1) appeared to be comprised mainly of equally sized animals. The second colony (colony #2), however, consisted of animals of different sizes, indicating different ages, or variation in exposure to hydrothermal fluid at different places within the colony.

Remotely operated vehicle assisted measurements of water temperature across and within the tubeworm colonies revealed a maximum recorded temperature of 19.4°C, about 5°C above ambient seawater temperatures of 14°C (Table 1). Diffusely venting warm hydrothermal fluids reached a maximum temperature of 58.4°C within small depressions in close proximity to the tubeworms. Chloride concentrations in these fluids were significantly higher than in ambient seawater indicating influx of hydrothermal brine eventually leading to the formation of small stratified brine pools in these depressions. Chemical analyses of vent and pore fluids sampled from sediment cores collected in the same area revealed that local hydrothermal fluids were anoxic, H_2_S-rich, acidic and displayed an elevated salinity. Concentrations of dissolved alkali and alkali earth elements (potassium, calcium, lithium, cesium), silica, arsenic, and tungsten (“Fluid end-member” in Table 3) were significantly higher when compared to normal bottom seawater. Fluid samples confirmed that this hydrothermal fluid was highly diluted with ambient seawater as it passed through the two tubeworm colonies leading to partly oxygenated waters (139 and 227 μmol/L dissolved O_2_, Table 3) and reduced levels of sulfide. Still, dissolved sulfide concentrations of 32 and 72 μmol/L were measured in water samples from among the tubes in the tubeworm colonies. In contrast, a maximum concentration of dissolved sulfide of 5,172 μmol/L was measured for the “hottest” hydrothermal fluids (58°C) sampled at Palinuro.

**Table 3:** **Composition of seawater-hydrothermal fluid mixtures inside tubeworm colonies #1 and #2**.

		Fluid endmember	Colony #1, *232 ROV-2*	Colony #2, *237 ROV-1*	Seawater *221 CTD*
T	°C		14.7	15.9	14.4
pH			6.9	6.7	8.2
Diss. O_2_	μM	–	230	139	
H_2_S	μM		72	32	–
Cl	mM	984	640	634	626
B	mM	11	0.52	0.52	0.47
Si	mM	1.7	0.02	0.02	0.01
Na	mM	681	528	526	520
K	mM	65	12	11.9	11.5
Ca	mM	78	12.4	12.3	11.8
Li	μM	7.7	0.06	0.06	0.03
Mn	μM		3	<1	<1
Fe	μM		<2	<2	<2
As	μM	222	9.6	10	<5
W	nM	224	1.6	–	<0.3

### Characterization of the tubeworms

#### Characterization of Palinuro tubeworms

**Figure 3:**
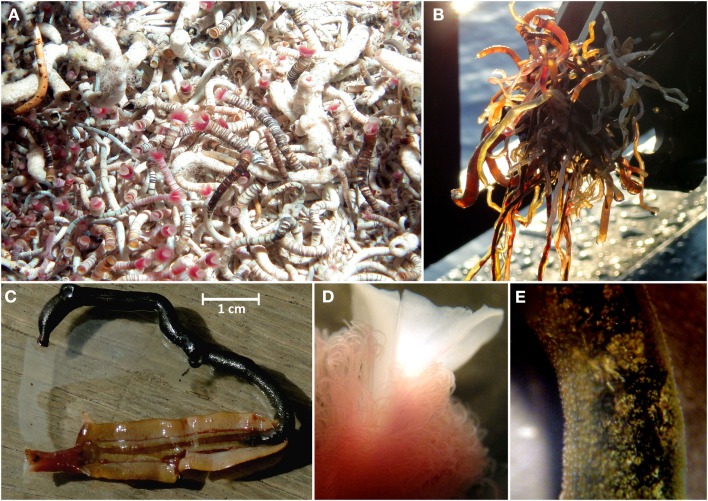
***Lamellibrachia* sp. tubeworms recovered from the Palinuro volcanic complex (Mediterranean Sea)**. **(A)** in their natural habitat (photo obtained at Palinuro during cruise Pos340), **(B)** directly after ROW Mohawk recovery (Pos412) onboard, **(C)** individual from colony #1 dissected from its tube (not used for further analysis), **(D)** stereo-micrograph of plume region, **(E)** stereo-micrograph of trophosome region.

A wide range of sizes of vestimentiferan tubeworms were collected from the Palinuro volcanic complex (Figure 3). The tubes of the collected animals ranged up to 15 cm in length with a maximum exterior diameter of 3 mm at the anterior end, decreasing slightly to the posterior end. The anterior region was banded reddish brown and white, whereas the posterior region was a more uniform brownish color. The tube walls were thick and rigid in the anterior region, becoming thinner and more flexible in the posterior regions. The vestimentiferan tubeworm hosts were identified by molecular analyses of the 18S rRNA gene as well as the mitochondrial genes for ribosomal 16S rRNA and the cytochrome c oxidase I (COI). All three genes were amplified from DNA extracted from tubeworm vestimentum, which is free of endosymbionts. Based on COI and mitochondrial 16S rRNA nucleotide analyses, all individuals analyzed from tubeworm colony #1 and the four individuals exhibiting different morphologies from tubeworm colony #2 obtained from the Palinuro volcanic complex belonged to the same species. The maximum difference between the COI gene fragments sequences was two bases (total investigated length 650 bp) and one base for the mitochondrial 16S rRNA gene (total investigated length 529 bp). In accordance with 18S rRNA gene, mitochondrial 16S rRNA gene and COI sequence the tubeworm could be identified as the newly described species *Lamellibrachia anaximandri* (Southward et al., 2011).

#### Characterization of Gulf of Mexico tubeworms

**Figure 4:**
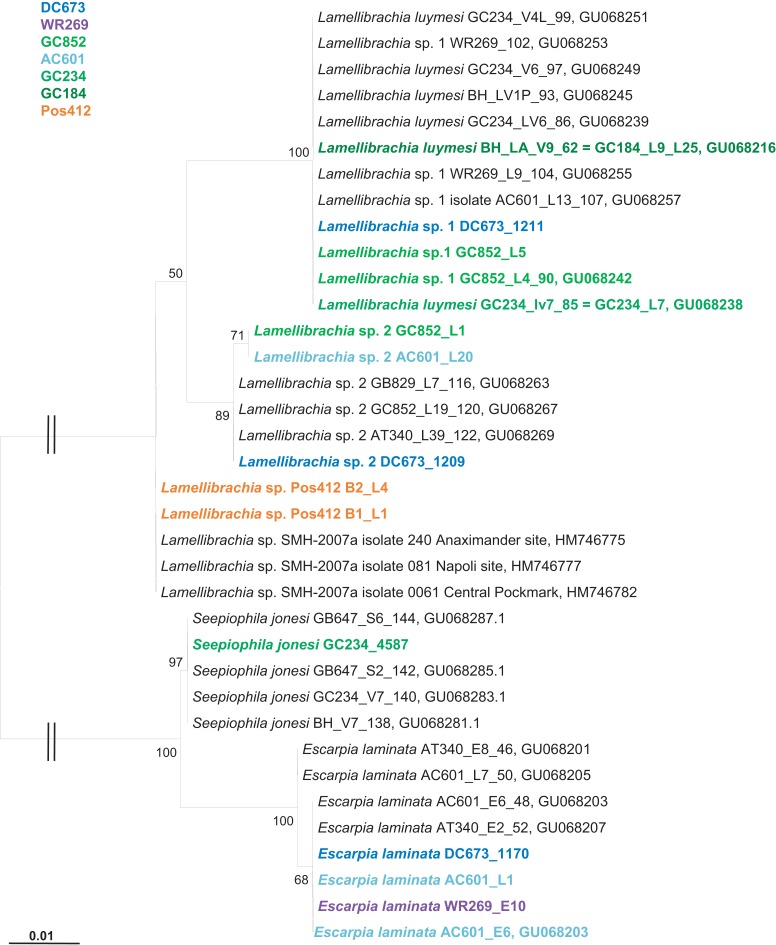
**Phylogenetic relationship of vestimentiferan tubeworms based on mitochondrial 16S rRNA gene sequences**. The Maximum Likelihood tree was calculated using the GTR model. Numbers at the nodes indicate the proportion of occurrences in 100 bootstrap replicates. The scale represents 0.01 substitutions per nucleotide site.

Gulf of Mexico tubeworm samples were identified using mitochondrial 16S rRNA genes amplified from DNA extracted from the endosymbiont free vestimentum tissue. Phylogenetic results confirmed the initial morphological characterizations of *Lamellibrachia luymesi*/*Lamellibrachia* sp. 1 (van der Land and Nørrevang, 1975), *Lamellibrachia* sp. 2, *Escarpia laminata* (Jones, 1985), and *Seepiophila jonesi* (Gardiner et al., 2001; Table 2; Figure 4).

### Characterization of the tubeworm endosymbionts

#### Endosymbionts of *L. anaximandri* from Palinuro

**Figure 5:**
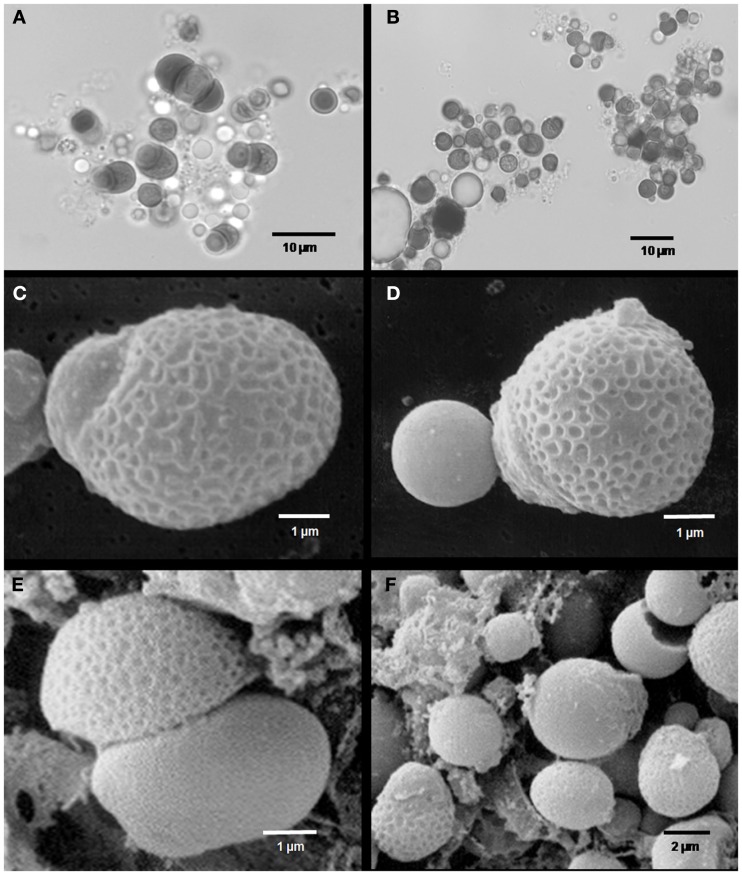
**Microscopic images of Palinuro *Lamellibrachia anaximandri* specimen endosymbionts**. **(A,B)** Light micrographs showing trophosome content with large spherical prokaryotic cells (dark) of different size and shape. Various stages of equal and unequal cell division as well as budding can be recognized. **(C–F)** Scanning electron micrographs showing endosymbionts with characteristically structured cell surface. Probable budding stages **(C,D)** and unequal cell division **(E,F)**.

Microscopic studies on the Palinuro *L. anaximandri* revealed high numbers of coccoid bacterial cells in broken trophosome tissue. These endosymbiotic bacterial cells varied considerably in size (2–10 μm diameter) and shape (spherical to irregularly coccoid). The color in the light microscope ranged from light to dark brown. Different modes of cell division were observed: equal division, unequal division, and budding (Figure 5). The cell surface of many endosymbionts showed a characteristic pattern of small invaginations (0.2–0.5 μm diameter), while others had a completely smooth surface. Frequently it was observed that cells in the process of budding or unequal cell division had a structured surface in the larger (older) part of the cell, while the bud was smooth (Figure 5).

**Figure 6:**
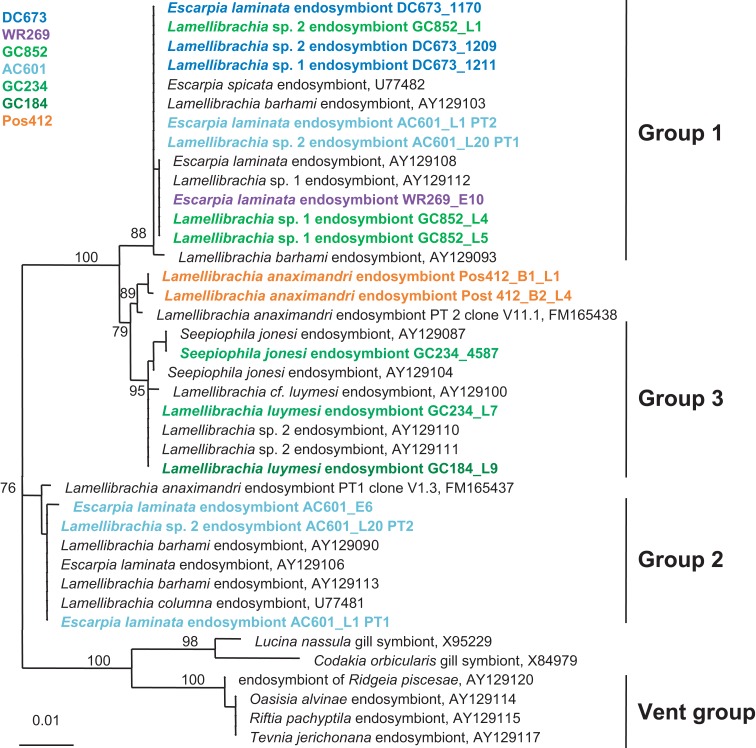
**Phylogenetic relationship of tubeworms endosymbionts based on 16S rRNA gene sequences**. The Maximum Likelihood tree was calculated using the GTR model. Numbers at the nodes indicate the proportion of occurrences in 100 bootstrap replicates. The scale represents 0.01 substitutions per nucleotide site.

The bacterial endosymbionts of the tubeworms were identified by constructing 16S rRNA gene clone libraries from DNA extracted from the trophosome tissue of 11 tubeworm individuals. For each specimen at least 20 clones were sequenced and analyzed. The bacterial 16S rRNA gene sequences of each specimen had >99% sequence identity, thus representing a single OTU. The consensus sequences (OTUs) from the different individuals were identical (100% sequence identity over a total length of 1,387 bp), indicating that only one bacterial endosymbiont phylotype was present in the Palinuro tubeworms. BLAST analysis revealed the gammaproteobacterial sulfide-oxidizing “phylotype 2” bacterial endosymbiont of *L. anaximandri* from the Eastern Mediterranean mud volcanoes as the closest relative (FM165438, 99.7% sequence identity, five nucleotides differences over a total length of 1,387 bp (Duperron et al., 2009). Other closely related sequences originate from other *Lamellibrachia* spp. and seep vestimentiferan endosymbionts from outside the Mediterranean (Figure 6). The endosymbionts of hydrothermal vent tubeworms *Riftia pachyptila* and *Tevnia jerichonana* were more distantly related and clustered on a separate branch within the 16S rRNA gene tree (Figure 6).

#### Phylogeny of seep vestimentiferan endosymbionts from the Gulf of Mexico

Bacterial endosymbionts from GoM tubeworm specimen were identified by amplification and direct sequencing of 16S rRNA genes from DNA extracted from the trophosome tissue.

The GoM vestimentiferan tubeworm’s endosymbionts were affiliated with the three monophyletic groups of seep vestimentiferan tubeworm endosymbiont sequences described by McMullin et al. (2003). Three specimens from GoM site DC673 (*Lamellibrachia* sp. 1, *Lamellibrachia* sp. 2, and *E. laminata* (DC673_1211, DC673_1209, DC673_1170) shared the identical (100% 16S rRNA gene sequence) “group 2” endosymbiont, very closely related to the sequences from *Lamellibrachia* sp. 1 and sp. 2 endosymbionts at site GC852 (GC852_L4, GC852_L1, GC852_L5) and *E. laminata* endosymbiont sequence from WR269 (WR269_E10). However, the endosymbiont sequence derived from an *E. laminata* specimen at site AC601 (AC601_E6) differed and clustered with “group 1” sequences. In two other tubeworm specimens (*E. laminata* and *Lamellibrachia* sp. 2) from site AC601 (AC601_L1, AC601_L20) we detected two different endosymbionts, one clustering with “group 1” (AC601_L1-PT1, AC601_L20-PT1) and the other with “group 2” (AC601_L1-PT2, AC601_L20-PT2). Endosymbiont sequences derived from *S. jonesi* and *L. luymesi* tubeworms from the shallower sites GC234 (GC234_4587, GC234_L7), and GC184 (GC184_L9) clustered with “group 3” sequences (Figure 6).

#### Genes involved in endosymbiont energy metabolism

In order to determine the potential energy-generating pathways for chemoautotrophic growth of the endosymbiont from the Palinuro *L. anaximandri*, we tried to amplify fragments of genes coding for key enzymes involved in the oxidation of sulfur compounds, hydrogen and methane.

**Figure 7:**
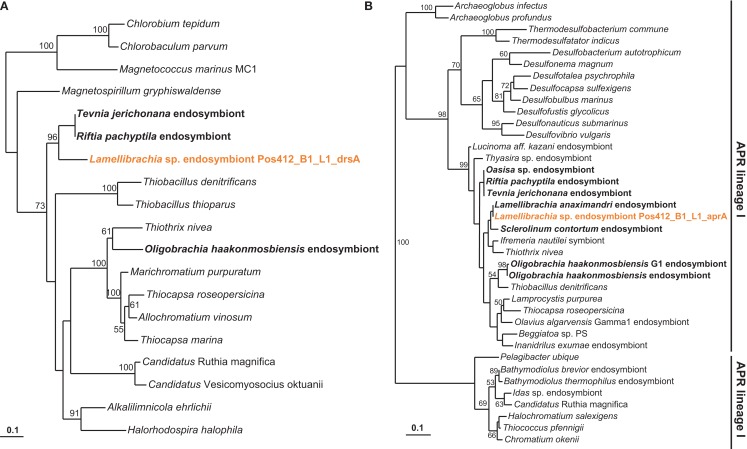
**Phylogenetic tree based on dsrA (A) and aprA (B) protein sequences**. The Maximum Likelihood tree was calculated using the JTT model. Numbers at the nodes indicate the proportion of occurrences in 100 bootstrap replicates. Sequences of tubeworm endosymbionts are depicted bold. Sequences obtained in this study are depicted colored. The scale represents 0.1 substitutions per amino acid position.

The genetic potential for sulfur oxidation of the endosymbiont was analyzed by amplifying gene fragments coding for dissimilatory sulfite reductase (*dsrAB*), APS reductase (*aprA*) – both enzymes of the APS pathway – and sulfate thiohydrolase (*soxB*), an essential component of the Sox multienzyme complex (Friedrich et al., 2001). Fragments of all three genes (*dsrAB, aprA*, *soxB*) were recovered supporting a sulfide-oxidizing chemotrophic energy metabolism of the endosymbiont. Sequence similarities as well as phylogenetic analysis showed them to be very similar to the endosymbionts of other *L*. *anaximandri* (*apr* within symbiont cluster) and the vestimentiferans *Riftia pachyptila* and *Tevnia jerichonana* from hydrothermal vents on the East Pacific Rise (*dsrAB*, *soxB*; Figure 7). The 397 bp *aprA* sequence showed highest similarity (97% nucleotide similarity, 100% amino acid similarity) to *L. anaximandri* endosymbiont “phylotype 1” described from seep specimens at the Amon mud volcano in the Eastern Mediterranean (Duperron et al., 2009). Phylogenetic analysis places the *Lamellibrachia* *aprA* sequences in a cluster of oxidizing lineage II APS reductase gene sequences of endosymbiotic and free-living beta- and gammaproteobacteria including endosymbionts of *Riftia* and *Tevnia* (Meyer and Kuever, 2007a; Markert et al., 2011; Gardebrecht et al., 2012). The *dsrAB* gene fragment (987 bp) was most closely related to dissimilatory sulfite reductase genes from the *Riftia*/*Tevnia* endosymbiont (NZ_AFZB01000023, EGW53672, and EGV52261, 80% nucleotide and 84% amino acid sequence similarity, Figure 7).

Likewise, the 986 bp *soxB* fragment from *L. anaximandri* from Palinuro showed highest similarities to *soxB* from *Candidatus* Endoriftia persephone (EF618617, EGV50931, and EGW54296, 84% nucleotide similarity, 90% amino acid similarity).

A fragment of the *hupL* gene, encoding the large subunit of a [NiFe] uptake hydrogenase was amplified using the primer set W1 and Wxy. BLAST search as well as phylogenetic analysis demonstrated highest similarity with reference sequences from the *Riftia*/*Tevnia* endosymbiont (EGV51840, EGW53439, 82% nucleotide identity, 93% amino acid identity). This enzyme has been shown to be involved in the oxidation of molecular hydrogen for energy generation (Petersen et al., 2011; Kleiner et al., 2012).

Key genes for enzymes of methane oxidation (*mxaF, mmoX, pmoA*) were not successfully amplified with the different primer sets (MxaF1003, MxaR1555, MxaR1561, mmoXA, mmoXB, A189F, MB661R; Costello and Lidstrom, 1999; Auman et al., 2000; Neufeld et al., 2007; Kalyuzhnaya et al., 2008) used in this study.

#### Genes involved in nitrate reduction

A nitric oxide reductase (*norCB*) gene sequence was successfully amplified and sequenced from the Palinuro *L. anaximandri* endosymbiont indicating the potential to reduce nitrate. The closest relative was again the endosymbiont of *Riftia*/*Tevnia* (EMBL entry ZP_08818090) with 95% amino acid sequence similarity. In the metagenomes of the *Riftia* and *Tevnia* endosymbionts, all genes needed for a complete respiration of nitrate to dinitrogen gas have been detected, and it has been suggested that the endosymbionts of these species could possibly use nitrate as alternative electron acceptor (Gardebrecht et al., 2012).

#### Genes involved in carbon fixation

To investigate the autotrophic potential of the endosymbionts, we tried to amplify key genes of two carbon fixation pathways, the CBB cycle and the reductive tricarboxylic acid (rTCA) cycle.

The CO_2_ fixing enzyme ribulose 1,5-bisphosphate carboxylase/oxygenase (RubisCO) is the key enzyme of the CBB cycle. In proteobacteria, two different types are known, form I, encoded by the *cbbL* gene, and form II encoded by *cbbM*. In accordance with other studies on *Lamellibrachia* spp. endosymbionts (Elsaied and Naganuma, 2001; Elsaied et al., 2002), we failed to amplify *cbbL* from the Palinuro tubeworm endosymbiont. In contrast, a fragment of the *cbbM* gene was detected supporting the usage of the CBB cycle for carbon fixation by the endosymbiont as was also previously demonstrated for other *Lamellibrachia* spp. endosymbionts (Elsaied and Naganuma, 2001; Elsaied et al., 2002; Vrijenhoek et al., 2007). The *cbbM* sequence displayed high similarity (96% nucleotide similarity, 100% amino acid similarity) to the bacterial endosymbiont of *L*. *anaximandri* from the Eastern Mediterranean (FM165442 and CAQ63473, Duperron et al., 2009), but was quite different from *cbbM* sequences of *Riftia/Tevnia* endosymbionts (AF047688, 78% nucleotide similarity, 75% amino acid similarity).

Based on genomic, proteomic, enzymatic as well as isotopic data, the *Riftia pachyptila* endosymbiont uses the rTCA cycle in addition to the CBB cycle for autotrophic carbon fixation (Markert et al., 2007, 2011; Gardebrecht et al., 2012). Yet a novel type of ATP citrate lyase (type II ACL) might be active in this case (Hügler and Sievert, 2011).

Newly designed primers were used to amplify a putative type II ACL from the endosymbionts of the Palinuro tubeworms. Unexpectedly, amplified fragments of this type II ACL gene indicate additional use of the rTCA cycle for carbon fixation in the Palinuro *L*. *anaximandri* endosymbiont as well. BLAST results with sequence similarities of 78% amino acid identities, as well as the phylogenetic analysis indicate the gene to be most closely related to the *Riftia* and *Tevnia* endosymbiont (NCBI entry ZP_08829917 and ZP_08817421). In contrast genes coding for a conventional ACL could not be detected using previously published primers for either subunits (*aclA* or *aclB*, Campbell et al., 2003; Hügler et al., 2005).

#### Carbon fixation genes in seep vestimentiferan endosymbionts from the Gulf of Mexico

**Figure 8:**
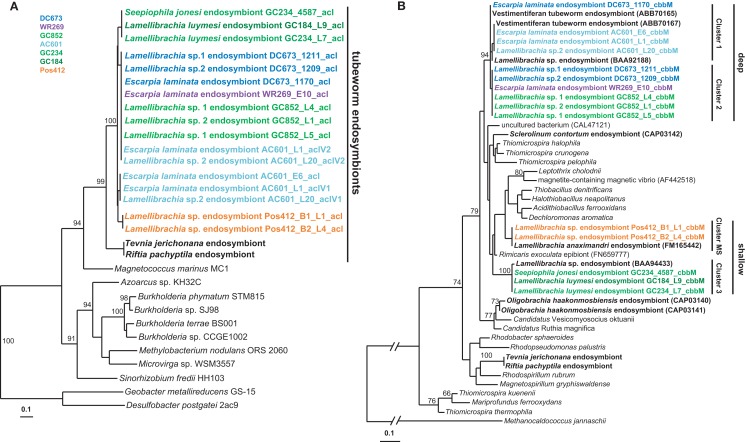
**Phylogenetic tree based on aclA (A) and cbbM (B) protein sequences**. The Maximum Likelihood tree was calculated using the JTT model. Numbers at the nodes indicate the proportion of occurrences in 100 bootstrap replicates. Sequences of tubeworm endosymbionts are depicted bold. Sequences obtained in the present study are depicted colored. The scale represents 0.1 substitutions per amino acid position.

The discovery of ACL genes in the *L*. *anaximandri* endosymbiont from the Palinuro volcanic complex raised the question about the further distribution of these genes in tubeworm endosymbionts, especially seep species. Thus we analyzed endosymbionts from 13 tubeworms from six different sites in the GoM. We discovered the type II ACL genes in all tubeworm endosymbionts investigated, regardless of their host species identity or site in the GoM (Table 2). Sequence analysis revealed the type II ACL gene to be highly conserved between the different GoM tubeworm endosymbionts. Three different phylotypes were found to be present in the 13 tubeworm samples in the GoM, and all three differed from the sequences found in the Palinuro *L. anaximandri* endosymbionts (Figure 8A). All GoM endosymbionts of host specimens from the sites DC673, WR269, and GC852 (DC673_1211, DC673_1209, DC673_1170, WR269_E10, GC852_L4, GC852_L1, GC852_L5) shared identical (100% AA similarity) type II ACL gene sequences (cluster 2) regardless of host species identity. Endosymbionts of GoM host specimens from the shallower Green Canyon sites GC234 and GC184 (GC234_4587, GC184_L9, GC234_L7) also bear one single type II ACL gene sequence (cluster 3; 100% AA similarity), which differed from the deep water site sequence in 14 AA. The third sequence type (cluster 1) was retrieved from endosymbionts of Alaminos Canyon site AC601 (AC601_E6, AC601_L1, AC601_L20). Within AC601_L1 and AC601_L20 a second ACL sequence type identical to the sequences of cluster 2 was also retrieved. In phylogenetic analysis, the GoM tubeworm endosymbiont ACL type II sequences formed a cluster together with the Mediterranean *L. anaximandri* endosymbiont sequences, and were clearly separated from the *Riftia*/*Tevnia* sequences (Figure 8A).

In addition to the *acl* genes, we also amplified the *cbbM* gene of the GoM tubeworm endosymbionts. As expected, all endosymbionts harbored a *cbbM* gene in addition to the *acl* gene. Similar to the 16S rRNA and *acl* genes, the *cbbM* genes from the GoM endosymbionts formed three different clusters (Figure 8B). Cluster 1 comprises the sequences of Alaminos Canyon site AC601 specimens, AC601_E6, AC601_L1, and AC601_L20. The *cbbM* sequences of DeSoto Canyon, Walker Ridge, and Green Canyon specimens DC673_1211, DC673_1209, WR269_E10, GC852_L4, GC852_L1, GC852_L5 form a second cluster (cluster 2), while the cbbM sequence of sample DC673_1170 falls in between these two clusters. Cluster 3 (Green Canyon samples GC234_4587, GC184_L9, GC234_L7) is clearly separated from the others. The *cbbM* sequences from the Mediterranean tubeworm endosymbionts form a separate cluster. Quite interestingly, the *cbbM* sequences of the *Riftia/Tevnia* endosymbionts are only distantly related (Figure 8B).

### Isotopic signature

Bulk organic carbon isotopes analyses of gill tissue from two Palinuro tubeworms resulted in δ^13^C values of −22.5 and −23.4‰, which are in accordance to previous measurements of Mediterranean *Lamellibrachia* spp. (Olu-Le Roy et al., 2004; Carlier et al., 2010) but more positive than most *Lamellibrachia* spp. from non-Mediterranean hydrocarbon seeps (Becker et al., 2011). The carbon isotopic composition of dissolved inorganic carbon in emanating diffuse fluids sampled at the tubeworm colonies display δ^13^C values of −0.39 and −0.68‰. The δ^13^C_DIC_ values of additional samples of shimmering water in the area range from −1.62 to +1.76‰.

## Discussion

### Phylogeny and biogeography of the Mediterranean tubeworms

The discovery of living vestimentiferan tubeworm colonies associated with active hydrothermal venting during a seafloor survey of the Palinuro volcanic complex (Mediterranean Sea) in July 2006 (Petersen et al., 2008; Monecke et al., 2009) came as a surprise, as until then, vent-associated tubeworms were only known from the Pacific Ocean. Living individuals of the tubeworms were sampled during a research cruise in 2011 and this communication is the first description of the worms and their endosymbionts. Phylogenetic analyses of 18S rRNA, COI and mitochondrial 16S rRNA genes showed that the tubeworms from Palinuro are *Lamellibrachia anaximandri* recently described from mud volcanoes of the Eastern Mediterranean (Southward et al., 2011). The highest *in vivo* temperatures measured among the tubes in tubeworm aggregations at the Palinuro hydrothermal vent field were 15.6–19.4°C, elevated by as much as 5.4°C from the surrounding Mediterranean Seawater (14°C) and the previously published tubeworm-bearing locations in the Eastern Mediterranean (13–14°C), extending the previously described temperature range of the species (Southward et al., 2011). *L. anaximandri* has also been detected in a mud volcano field in the Western Mediterranean (Hilário et al., 2011), as well as on two ship wrecks in the Eastern Mediterranean (110 km southeast of Crete, Hughes and Crawford, 2008) and the Southern Tyrrhenian Sea (Gambi et al., 2011; Figure 1). Even though this species has not been detected in the Northeastern Atlantic it has been hypothesized to occur at the West African and Lusitanian margins as well (Hilário et al., 2011).

The high diversity of habitats for the Mediterranean *Lamellibrachia* species is in accordance with *Escarpia* spp. and other *Lamellibrachia* spp. Originally regarded as seep species, they were subsequently found in several non-seep habitats, i.e., at sediment covered hydrothermal sites in the Pacific (Juniper et al., 1992; Fujikura et al., 2006; Miake et al., 2006; Miura and Kojima, 2006), as well as ship wrecks (Dando et al., 1992), and whale falls (Feldman et al., 1998). Considering the high diversity of so-called “seep” tubeworm habitats a high site-flexibility of these organisms becomes apparent and supports the importance of different non-seep habitats in their geographic distribution and the stepping stone hypothesis (Kimura and Weiss, 1964; Smith and Kukert, 1989; Black et al., 1994; Olu et al., 2010). Larval survival of at least three weeks and about five weeks has been demonstrated for the vestimentiferans *Riftia pachyptila* and *Lamellibrachia luymesi* respectively, suggesting potential dispersal distances on the order of 100 km for seep and vent vestimentiferans (Young et al., 1996; Marsh et al., 2001; Tyler and Young, 2003). A variety of reducing habitats, functioning as dispersal stepping-stones separated by days or weeks and connected by currents or shared water masses could facilitate the large species ranges described for many vestimentiferans, including the seep species *L. barhami*, which has been found in seep and low activity vent sites spanning at least 4,000–6,000 km of geographical distance (McMullin et al., 2003).

### Phylogeny of endosymbionts

The trophosome of the *L. anaximandri* specimens from Palinuro analyzed in this study harbored a single gammaproteobacterial phylotype regardless of collection site or morphotype. The endosymbiont was closely related (99.7%) to the phylotype 2 found in *L. anaximandri* from the Amon mud volcano east of the Nile deep-sea fan (Duperron et al., 2009). The dominating endosymbiont (phylotype 1) of the seep specimen from the Amon mud volcano was not found in the tubeworms at the Palinuro hydrothermal vents.

This study is the first to characterize vestimentiferan tubeworm endosymbionts of shallow hydrothermal vents in the Mediterranean Sea. The gammaproteobacterial endosymbiont clusters with endosymbionts of other seep-associated tubeworms and are clearly distinct from the endosymbionts of vent tubeworms like *Riftia* and *Tevnia* (Fisher et al., 1997; Nelson and Fisher, 2000; McMullin et al., 2003; Vrijenhoek et al., 2007). The phylogenetic affiliation of the Palinuro *L. anaximandri* endosymbiont with “group 3” endosymbiont 16S rRNA gene sequences, as well as the affiliation of the dominating phylotype of *L. anaximandri* specimen obtained from 1,157 m depth at Amon mud volcano with “group 1,” may indicate separation by depth, as suggested for other seep vestimentiferan endosymbionts (McMullin et al., 2003). However, both Mediterranean phylotype sequences show considerable differences to the “group 1” and “group 3” cluster sequences in signature nucleotide positions (Table A1 in Appendix) and Mediterranean and GoM tubeworms do not share identical endosymbiont phylotypes. Further, in the Amon mud volcano specimen, phylotypes of “group 1” and “group 3” are present, yet in assumed different abundances (deduced from the numbers of sequences in the clone libraries; Duperron et al., 2009). Endosymbionts of different groups are also present in individuals of *Lamellibrachia* sp. 2 (AC601_L20) and *E. laminata* (AC601_L1) tubeworms from the GoM site AC601 (this study). Thus separation by depth alone cannot explain these observations and further studies are needed in order to reveal the question how endosymbionts are selected by their vestimentiferan hosts.

### Metabolic characteristics of the endosymbiont

Based on the functional gene analyses of this study, the endosymbiont of *Lamellibrachia anaximandri* from Palinuro is a sulfide-oxidizing chemoautotroph. δ^13^C values measured in this study are in consistence with previous studies of *L. anaximandri* from Eastern Mediterranean mud volcano fields (Olu-Le Roy et al., 2004; Carlier et al., 2010), and together with delta δ^15^N and δ^34^S from previous studies support a chemoautotrophic endosymbiont based nutrition for the host tubeworm (Carlier et al., 2010). Due to the presence of *dsrAB* and *aprA* genes, sulfide oxidation most likely is carried out via the APS pathway with sulfite and adenosine phosphosulfate as intermediates. As in the *Riftia pachyptila* endosymbiont, *soxB* is also present in the endosymbiont of *L. anaximandri* from Palinuro. In thiosulfate-utilizing bacteria, SoxB functions as sulfate thiohydrolase. However, since tubeworm endosymbiont carbon fixation is not stimulated by thiosulfate, its function in the tubeworm endosymbionts remains uncertain (Fisher et al., 1989; Markert et al., 2011).

Although high methane fluxes were noted in the habitat of *L. anaximandri* in mud volcano habitats (Olu-Le Roy et al., 2004), genes of methane oxidation were not successfully amplified with the primer sets used in this study. In contrast, the potential to use hydrogen as an energy source was suggested by the detection of the key gene for hydrogen oxidation, *hupL* in the endosymbiont from the Palinuro *L. anaximandri*. The *hupL* gene was most similar to the respective genes of the *Riftia* and *Tevnia* endosymbionts, where it is even expressed *in situ* (Markert et al., 2011; Petersen et al., 2011). Hydrogen concentrations have not been measured in the hydrothermal fluids from Palinuro volcano complex. However, hydrogen is present in the fluids at many hydrothermal vents, and elevated hydrogen contents are present at vent systems associated with ultramafic (mantle) rocks, or, e.g., following a volcanic eruption (Wetzel and Shock, 2000; Allen and Seyfried, 2003; Kumagai et al., 2008; Petersen et al., 2011). Such H_2_-rich fluids found at vent systems associated with ultramafic rocks have recently been shown to be used as an energy source by the endosymbionts of a mussel, *Bathymodiolus puteoserpentis* (Petersen et al., 2011). The endosymbionts in *Bathymodiolus* spp. mussels are located on the external edge of the cells of gill filaments that are themselves only two cells thick. As a result, passive diffusion of the energy source (hydrogen, sulfide, and/or methane in different species) is sufficient to fuel the chemoautotrophic life style of these animals (Childress and Fisher, 1992). In contrast, the endosymbionts in tubeworms are deep in an interior tissue and must rely on the host blood to supply the electron donor for chemoautotrophy. Sulfide is transported in millimolar concentrations to the trophosome, bound to hemoglobin molecules in vestimentiferan tubeworms. Transport molecules for hydrogen or methane have not been found in these animals and thus both hydrogen and methane are unlikely to contribute significantly to the metabolism of the intact symbiosis in most environments (Childress and Fisher, 1992). However, the gammaproteobacterial endosymbiont might use hydrogen as potential energy source in its free-living stage. Thus, the potential for use of hydrogen by tubeworm endosymbionts deserves additional study.

Detection of *cbbM* sequences coding for a form II RubisCO in all vestimentiferan tubeworms reviewed in this study indicates that the potential to fix CO_2_ via the CBB cycle is widespread in vestimentiferan tubeworms (Elsaied and Naganuma, 2001; Elsaied et al., 2002; Naganuma et al., 2005; Vrijenhoek et al., 2007; Duperron et al., 2009) and the detection of all genes of this cycle in the metagenome of the *Riftia* endosymbiont suggest this pathway is fully functional in vestimentiferans (see Markert et al., 2011 for further details). Enzyme activity measurements of RubisCO and phosphoribulokinase add further evidence to the usage of the CBB cycle in *Riftia* and *Lamellibrachia* endosymbionts (Felbeck, 1981; Felbeck et al., 1981). Up to now, there is no evidence for the presence of RubisCO form I (*cbbL*) in either the *Riftia/Tevnia* or any *Lamellibrachia* endosymbiont (this study; Elsaied and Naganuma, 2001; Elsaied et al., 2002; Naganuma et al., 2005; Duperron et al., 2009).

In addition, *acl* genes, coding for ATP citrate lyase, the key enzyme of the rTCA cycle were recovered from the Palinuro *L. anaximandri* endosymbiont using newly designed primers, suggesting the presence of the rTCA cycle as alternate carbon fixation pathway. The operation of the rTCA cycle in addition to the CBB cycle was first shown for the *Riftia* and *Tevnia* endosymbiont using a combination of metagenomic, proteomic and enzymatic approaches (Markert et al., 2007; Robidart et al., 2008; Gardebrecht et al., 2012). In the case of the *Riftia/Tevnia* endosymbiont, citrate cleavage is accomplished by an unusual type of ATP citrate lyase, tentatively named ACL type II (Hügler and Sievert, 2011). The recovered *acl* sequence from the Mediterranean Palinuro *L. anaximandri* endosymbiont showed high similarities to the sequence of the *Riftia*/*Tevnia* endosymbiont (Figure 8A). Subsequent analyses of seep vestimentiferan (*Escarpia, Seepiophila*, and *Lamellibrachia*) endosymbionts from different sites at the Gulf of Mexico showed the presence of type II ACL genes there as well. This implicates a wider distribution of these genes than previously thought. The presence of two different carbon fixation pathways – the CBB cycle and the rTCA cycle – in a single bacterium seems not restricted to the *Riftia*/*Tevnia* endosymbiont, but rather seems to be a common feature of vestimentiferan tubeworm endosymbionts, regardless of genus or habitat.

Despite the still rather scarce dataset of type II ACL gene sequences, these sequences appear to be monophyletic in tubeworm endosymbionts (Figure 8A). Similarly, ribosomal genes as well as *apr*A genes support a monophyletic origin for the tubeworm endosymbionts (Figures 6 and 7). In contrast, a monophyletic origin of the *cbbM* gene of tubeworm endosymbionts is not clearly supported by the phylogenetic analyses performed here (Figure 8B). This could mean that the rTCA cycle is the evolutionary older CO_2_ fixation pathway in the endosymbionts, and the *cbbM* gene is acquired afterward, e.g., via lateral gene transfer. This evolutionary aspect clearly requires further studies.

The presence of two different carbon fixation pathways increases the metabolic versatility of the tubeworm endosymbionts. In case of the *Riftia/Tevnia* endosymbiont proteomic data suggest the usage of both pathways simultaneously (Markert et al., 2007, 2011; Gardebrecht et al., 2012). This is also supported by the isotopic signature of the *Riftia* tubeworms (Markert et al., 2007). The carbon isotopic fractionation associated with the rTCA cycle is generally smaller than the one observed for the CBB cycle (House et al., 2003). Considering the carbon isotopic composition of ca. −23‰ measured for plume tissue of two *L. anaximandri* tubeworms from Palinuro low temperature diffuse vent sites and a respective carbon isotopic composition of dissolved inorganic carbon (δ^13^C_DIC_ between −0.7 and −0.4‰), the isotopic difference would be consistent with the operation of the CBB cycle for autotrophic carbon fixation. Neither a greatly attenuated isotopic fractionation characteristic for the rTCA cycle nor an isotopic signature reflecting a substantial contribution from methane-derived carbon is discernible at the site studied here. Yet, one has to keep in mind, that the isotopic signature provides only indirect evidence and neither the actual fractionation by the enzymes involved in the rTCA cycle present in tubeworm endosymbionts, nor fractionation during uptake and transport of DIC to the endosymbionts are known. Thus future studies are needed in order to determine the conditions for the usage, as well as the regulation of the two different carbon fixation pathways in vestimentiferan tubeworm endosymbionts (Hügler and Sievert, 2011).

## Conclusion

In this study we characterize vestimentiferan tubeworms and their endosymbionts from the Mediterranean Sea and the GoM. The tubeworms retrieved from a shallow water hydrothermal vent field in the Western Mediterranean – Palinuro volcanic complex – represent the first vestimentiferan tubeworms found associated with hydrothermal venting outside the Pacific Ocean. Our molecular studies of marker genes (18S rRNA, mitochondrial 16S rRNA, and COI) identify the tubeworms as the recently described species *L. anaximandri*, the only vestimentiferan species described from the Mediterranean Sea to date.

Based on 16S rRNA gene surveys we conclude that the Palinuro *L. anaximandri* harbor a single gammaproteobacterial endosymbiont, closely related to endosymbionts of other *Lamellibrachia* spp. (Figure 6). Carbon isotopic data and the analysis of functional genes suggest a sulfide-oxidizing chemoautotrophic lifestyle. Energy can be generated by oxidizing reduced sulfur compounds via the APS pathway involving dissimilatory sulfite reductase and APS reductase. Due to the presence of a *hupL* gene one can speculate that the endosymbiont has the potential to use hydrogen as a supplemental energy source. Nitrate could potentially serve as alternative electron acceptor for the endosymbiont, as we detected a nitric oxide reductase gene sequence (*norCB*) and it was shown, that the metagenome of the *Riftia*/*Tevnia* endosymbiont includes all genes needed for the complete reduction of nitrate to dinitrogen gas (Gardebrecht et al., 2012).

Surprisingly, we were able to detect the key genes of two alternative carbon fixation pathways, namely *cbbM*, encoding RubisCO form II, the key enzyme of the CBB cycle, and a gene coding for ATP citrate lyase type II, the key enzyme of the rTCA cycle. Newly designed primers were used to amplify a gene sequence of the type II ACL. The presence of the rTCA cycle in addition to the CBB cycle for carbon fixation was previously shown for the endosymbiont of the vent-associated tubeworms *Riftia pachyptila* and *Tevnia jerichonana* (Markert et al., 2007; Gardebrecht et al., 2012). However, before this study, only the CBB cycle was documented as a carbon fixation pathway for *Lamellibrachia* spp. (Felbeck, 1981; Felbeck et al., 1981; Elsaied and Naganuma, 2001; Elsaied et al., 2002; Vrijenhoek et al., 2007). We also demonstrate the presence of the key genes of both carbon cycles in the endosymbionts from *Lamellibrachia luymesi, Lamellibrachia* sp. 1*, Lamellibrachia* sp. 2, *Escarpia laminata*, and *Seepiophila jonesi* from the Gulf of Mexico. These results suggest that the occurrence of two carbon fixation pathways in one bacterium may be a common feature of vestimentiferan tubeworm endosymbionts, which in turn indicates that this feature is more widely distributed than previously considered. It has already been shown, that carbon fixation through the rTCA cycle is important at deep-sea hydrothermal vent sites (cf. Hügler and Sievert, 2011 and references therein). Our study indicates that the rTCA cycle could play an important role at seep sites as well.

## Conflict of Interest Statement

The authors declare that the research was conducted in the absence of any commercial or financial relationships that could be construed as a potential conflict of interest.

## Appendix

**Table A1:** **Signature nucleotide of tubeworm endosymbiont 16S rRNA gene sequences from vent- and seep vestimentiferan tubeworms**.

***E. coli* pos**.	**75**	**77**	**79**	**80**	**90**	**92**	**137**	**231**	**250**	**258**	**268**	**286**	**301**	**381**
Vent	c	g	a	g	t	t	c	t	a	g	c	a	g/a	a/c
Seep-1	a	t	g	a	c	a	t	c	t	a	t	g	g	c
Seep-2	a	t	g	a	c	a	t	c	t	a	t	g	g	c
Seep-3	a	t	g	a	c	a	t	c	t	a	t	g	g	c
PT-1	a	t	g	a	c	a	t	c	a	a	c	g	g	c
PT-2	a	t	g	a	c	a	t	c	t	a	t	g	g	a
Pos412	a	t	g	a	c	a	t	c	t	a	t	g	g	c

***E. coli* pos**.	**441**	**446**	**457**	**459**	**460**	**461**	**463**	**469**	**488**	**646**	**648**	**650**	**658**	**831**

Vent	a	g	c	g	a	g	a	c	c	g	a	g	a	c
Seep-1	a	t	c	a	g	a	t	g	a	c	t	a	g	t
Seep-2	a	t	t	a	g	a	t	g	a	c	t	a	g	t
Seep-3	g	t	c	a	g	g	t	a	a	c	t	a	g	t
PT-1	a	t	c	a	g	g	t	a	a	c	t	a	g	t
PT-2	a	t	c	a	g	a	t	a	a	c	t	a	g	t
Pos412	a	t	c	a	g	a	t	a	a	c	t	a	g	t

***E. coli* pos**.	**849**	**859**	**986**	**987**	**998**	**999**	**1000**	**1002**	**1006**	**1007**	**1008**	**1009**	**1010**	**1011**

Vent	c	c	a	g	c	c	t	g	c	t	t	t	c	t
Seep-1	t	a	t	a	c	c	t	a	c	t	t	g	t	t
Seep-2	t	a	t	a	t	g	a	a	t	c	c	t	g	t
Seep-3	t	a	t	a	t	g	a	a	t	c	c	a	g	t
PT-1	t	a	t	a	c	c	t	a	c	t	t	g	t	t
PT-2	t	a	t	a	t	g	a	a	t	c	c	a	g	c
Pos412	t	a	t	a	t	g	a	a	t	c	c	a	g	c

***E. coli* pos**.	**1019**	**1020**	**1021**	**1022**	**1023**	**1036**	**1037**	**1040**	**1041**	**1042**	**1043**	**1121**	**1135**	**1152**

Vent	g	a	t	t	g	g	c	a	g	t	g	t	g	a
Seep-1	a	c	t	t	g	a	t	a	g	t	g	a	g	t
Seep-2	c	g	g	g	a	–	c	g	a	a	a	t	a	a
Seep-3	c	a	g	g	a	–	c	g	a	a	a	a	g	t
PT-1	a	c	t	t	g	a	t	a	g	t	g	a	g	t
PT-2	c	a	g	g	a	–	c	g	a	a	a	a	g	t
Pos412	c	a	g	g	a	–	c	g	a	a	a	a	g	t

***E. coli* pos**.	**1155**	**1219**	**1243**	**1257**	**1278**									

Vent	g	t	c	t	c									
Seep-1	a	a	c	c	t									
Seep-2	a	a	a/c	t	t									
Seep-3	a	a	c	c	t									
PT-1	a	a	c	c	t									
PT-2	a	a	c	c	t									
Pos412	a	a	c	c	t									

*“Vent” vestimentiferan group includes endosymbiont rRNA gene sequences of *Riftia pachyptila*, *Tevnia**jerichonana*, and *Osasisia alvinae*. “Seep” vestimentiferan tubeworm endosymbiont cluster “seep-(1–3)” refer to grouping according to McMullin et al. (2003). PT1 and PT2 refer to *Lamellibrachia anaximandri* endosymbionts, phylotype 1 and phylotype 2 according to Duperron et al. (2009). Pos412 refers to the *Lamellibrachia anaximandri* endosymbiont rRNA gene sequence phylotype obtained from the Palinuro volcanic complex during cruise Pos412 of R/V *Poseidon* in this study. The position numbers refer to *E. coli* gene sequence positions*.
